# Feasibility of 3-dimensional video-assisted thoracic surgery (3D-VATS) for pulmonary resection

**DOI:** 10.1186/s13022-015-0018-x

**Published:** 2015-10-15

**Authors:** Chris Dickhoff, Wilson W. Li, Petr Symersky, Koen J. Hartemink

**Affiliations:** Department of Cardio-Thoracic Surgery, VU University Medical Center, P.O. Box 7057, 1007 MB Amsterdam, The Netherlands; Department of Surgery, VU University Medical Center, P.O. Box 7057, 1007 MB Amsterdam, The Netherlands; Department of Surgery, Netherlands Cancer Institute – Antoni van Leeuwenhoek Hospital, P.O. Box 90203, 1006 BE Amsterdam, The Netherlands

**Keywords:** 3-Dimensional, VATS, Robot, Technique

## Abstract

**Background:**

Two-dimensional video-assisted thoracic surgery (2D-VATS) has gained its position in daily practise. Although very useful, its two-dimensional view has its drawbacks when performing pulmonary resections. We report our first experience with 3-dimensional video-assisted surgery (3D-VATS). Advantages and differences with 2D-VATS and robotic surgery (RS) are discussed.

**Methods:**

To evaluate feasibility, we scheduled patients for surgery by 3D-VATS who would normally be treated with 2D-VATS. The main difference of the equipment in 3D-VATS compared with former VATS equipment, is the flexible camera-tip (100-degrees) and the necessary 3D-glasses.

**Results:**

Four patients were successfully operated for anatomic pulmonary resections. On-the-structure dissection was easily performed and with the flexible camera-tip, a perfect view can be obtained, with clear visualisation of important (hilar) structures. These features highly facilitate the surgeon in tissue preparation and recognition of the dissection planes.

**Conclusion:**

In our opinion, 3D-VATS is superior to 2D-VATS for performing anatomic pulmonary resection and we expect an improvement in terms of operation time and learning curve. Furthermore, it is a valuable alternative for RS at lower costs.

## Background

In the search for lower morbidity in thoracic surgery, alternatives for invasive thoracotomies, like the mini-thoracotomy and video-assisted thoracic surgery (VATS) techniques, were developed. The latter technique has proven to be feasible and safe, even in the setting of neoadjuvant therapies [1]. Furthermore, results in terms of survival are at least equal when compared with pulmonary resection for cancer via thoracotomy, but with less morbidity [2]. However, there are certain disadvantages when using VATS: there is a steep learning curve, poorer optics and visualisation of the hilar structures and dissection planes because of only two-dimensional visualization [3]. The robot has 3-dimensional (3D), magnified intrathoracic view and is equipped with smaller instruments having more degrees of freedom [4]. This results in a more natural way of performing pulmonary resections and reduces length of stay, peroperative blood loss, and even reduces 30-day mortality [3], with oncologic results being comparable with thoracotomy and VATS [5]. Although robotic pulmonary resection is more expensive than VATS, which is mainly due to capital depreciation and the cost of robot-specific supplies/equipment [6], these costs might drop to a level comparable with the costs of VATS instruments as a result of wider implementation of the technique in the near future.

Recently, the 3D-VATS was introduced, which potentially combines the advantages of VATS and robotic surgery. To our knowledge, this is the first report describing the features and feasibility of the 3D-VATS for anatomic pulmonary resections.

## Methods

The study was approved by our institutional medical ethics committee without the need for written informed consent. Patients were staged according to the 7th edition of the IASLC staging of lung cancer [7]. After staging, none of the patients had neoadjuvant therapy, and all were planned for surgical resection by lobectomy after being discussed in our multidisciplinary tumor board meeting.

**Fig. 1:**
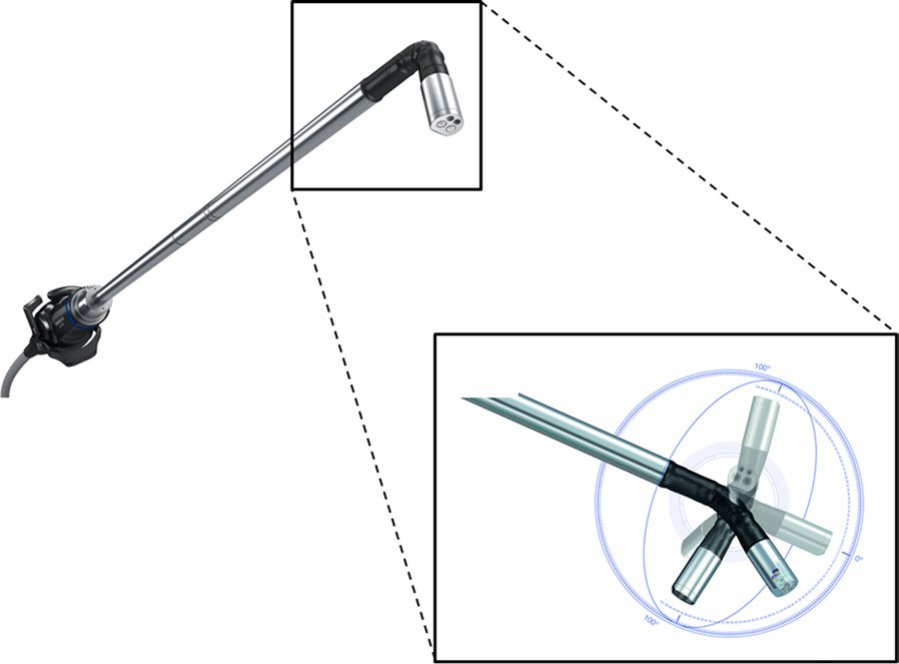
Illustration of the 3D-camera with flexible tip used during 3D-VATS anatomic pulmonary resection

3D-VATS combines 3D optics with a 100-degree angulating endoscope with high range of motion (Fig. 1), with proper visualisation of the hilar structures and anatomic dissection planes as a result. 3-dimensional spectacles are part of the equipment and worn by all personnel involved in the operation. The system is equipped with an HD-hard disk drive to record the procedures in both 2D and 3D, facilitating teaching and presentation purposes. All personnel involved in the surgical procedure wore 3D-spectacles. There is no need to put off the glasses during the procedure when not using the 3D-camera, for example in the beginning of the surgical procedure during first port placement, because visualisation is undisturbed through the glasses. Once the camera is in use and dissection of pulmonary structures begins, the 3D-view and the flexible tip of the camera reveals its advantages.

## Results

The 3D-VATS technique was used in four patients, all female: two patients underwent 3D-VATS lobectomy for NSCLC, one for pulmonary paraganglioma and one patient had a segmentectomy because of a cavitating nodule and was planned for high-dose steroids because of vasculitis (segment 6, right lower lobe). Patient positioning and surgical preparation did not differ from the 2D-VATS technique. Furthermore, port placement was the same as in 2D-VATS when performing an anatomical resection. However, one should be aware not to position the camera-port too cranial, because of articulating part of the flexible camera should be intra-thoracic and the tip should be positioned not to close to the working field in order to obtain the best optical performance by the camera. There was no major blood loss during surgery and no technical problems occurred. Postoperative recovery was uneventful in all patients.

## Discussion

To our knowledge, this is the first report about 3D-VATS for (anatomic) pulmonary resections. However we have only a few patients treated, its benefits are clear. First of all, the 3D-camera gives excellent visualisation of the anatomical planes and structures which enables proper on-the-structure dissection. Although not properly studied thus far, we think this will reduce operating time and might shorten the learning curve for VATS procedures. Furthermore, when compared with 2D-VATS, the surgical technique is the same in 3D-VATS and thus there is no need to go through a new learning curve when the surgeon is familiar with VATS already (which indeed is the case with robotic surgery). For trainees or surgeons without experience with the VATS technique, the learning curve will be shorter and less steep; the angulation of the tip of the camera to a maximum of 100 degrees highly facilitates dissection of the major vessels (e.g. superior pulmonary artery trunk in right upper lobectomy) and positioning the stapler in completing the fissures. Lymph node dissection is facilitated by both the flexible tip and the 3D-view, in the hilum and fissures when preparing on the vessels and bronchus, but also in the mediastinal lymph node stations. The use of 3D vision has already been demonstrated in randomized studies to improve the learning curve and shorten task performance times for laparoscopic surgery for both novice trainees [8] as well as experienced surgeons [9].

With the introduction of the robotic anatomical pulmonary resection, the question rises whether there is place for adapting the 3D-VATS technique. From a surgical point of view, using the robot is more ergonomic due to its in-line-axis with the surgeon, sitting position, head- and arm support, excellent 3D-view and a superb range of motion for all instruments [3]. However, the 3D-VATS technique has comparable view on the operation field, plus the advantage of the flexible camera tip which enables the looking-around-the-corner-view. Surgeons confident with the 2D-VATS technique and equipment will adapt this technique without need for extra training, which is a great advantage when compared with use of the robot. The costs for 3D-VATS equipment do not substantially differ from that of 2D-VATS. This can be important from a hospital point-of-view in the current era of reducing expenses on health-care; the higher cost of robotic surgery when compared with both open (thoracotomy) and 2D-VATS [6] might be a reason to adapt the 3D-technique and not the robotic technique. It is only after wide adaption of the latter, that the robotic instruments and purchase price will diminish, probably to levels comparable to that of VATS instruments.

## Conclusion

In our opinion, 3D-VATS is promising in terms of feasibility and implementation in daily (thoracic) surgical practise. The hypothesis that it will reduce learning curve and operation time for anatomic pulmonary resection, merits further evaluation in prospective trials.
